# Population genetic analyses unveiled genetic stratification and differential natural selection signatures across the *G*-gene of viral hemorrhagic septicemia virus

**DOI:** 10.3389/fgene.2022.982527

**Published:** 2022-12-12

**Authors:** Kiran Nigar, Sehrish Kakakhel, Asifullah Khan, Hizbullah Khan, Komal Zaib, Shaoqing Wen

**Affiliations:** ^1^ Department of Biochemistry, Abdul Wali Khan University Mardan, Mardan, Pakistan; ^2^ CAS Key Laboratory of Molecular Virology and Immunology, The Center for Microbes, Development and Health, Institut Pasteur of Shanghai, Chinese Academy of Sciences, Shanghai, China; ^3^ University of Chinese Academy of Sciences, Beijing, China; ^4^ Ministry of Education Key Laboratory of Contemporary Anthropology, Department of Anthropology and Human Genetics, School of Life Sciences, Fudan University, Shanghai, China; ^5^ Institute of Archaeological Science, Fudan University, Shanghai, China

**Keywords:** viral hemorrhagic septicemia virus, population genetics, phylogenetic, selection pressure, evolutionary distinction

## Abstract

**Introduction:** Viral hemorrhagic septicemia virus (VHSV) is the most lethal pathogen in aquaculture, infecting more than 140 fish species in marine, estuarine, and freshwater environments. Viral hemorrhagic septicemia virus is an enveloped RNA virus that belongs to the family Rhabdoviridae and the genus *Novirhabdovirus*. The current study is designed to infer the worldwide Viral hemorrhagic septicemia virus isolates’ genetic diversity and evolutionary dynamics based on *G*-gene sequences.

**Methods:** The complete *G*-gene sequences of viral hemorrhagic septicemia virus were retrieved from the public repositories with known timing and geography details. Pairwise statistical analysis was performed using Arlequin. The Bayesian model-based approach implemented in STRUCTURE software was used to investigate the population genetic structure, and the phylogenetic tree was constructed using MEGA X and IQ-TREE. The natural selection analysis was assessed using different statistical approaches, including IFEL, MEME, and SLAC.

**Results and Discussion:** The global Viral hemorrhagic septicemia virus samples are stratified into five genetically distinct subpopulations. The STRUCTURE analysis unveiled spatial clustering of genotype Ia into two distinct clusters at K = 3. However, at K = 5, the genotype Ia samples, deposited from Denmark, showed temporal distribution into two groups. The analyses unveiled that the genotype Ia samples stratified into subpopulations possibly based on spatiotemporal distribution. Several viral hemorrhagic septicemia virus samples are characterized as genetically admixed or recombinant. In addition, differential or subpopulation cluster-specific natural selection signatures were identified across the *G*-gene codon sites among the viral hemorrhagic septicemia virus isolates. Evidence of low recombination events elucidates that genetic mutations and positive selection events have possibly driven the observed genetic stratification of viral hemorrhagic septicemia virus samples.

## 1 Introduction

Viral hemorrhagic septicemia virus (VHSV) belongs to the order Mononegavirales, the family Rhabdoviridae, and the genus *Novirhabdovirus* (Fauquet et al., 2005). VHSV is a rapidly evolving RNA virus, with mutation rates ranging from µ = 10^−4^ to 10^−2^ nucleotide substitutions per site per year (Pierce and Stepien, 2012). The small genomes, short generation times, rapid mutation, and lack of polymerase proof-reading make the RNA viruses evolve quickly and diversify rapidly (Elena and Sanjuán, 2005). The higher genetic diversity increases the viral population’s fitness to rapidly spread to new hosts (Stepien et al., 2015). The VHSV infection induces 90% cumulative mortality and is considered the most devastating pathogen in the aquaculture industry. VHSV is readily transmitted during fish congregation in the spring spawning season at 9–12°C. The infection leads to erratic swimming behavior, exophthalmia (bulging eyes), bloated abdomen, and extensive external/internal bleeding, resulting in liver and kidney damage (Winton and Einer-Jensen, 2002; Daniels and Watanabe, 2010). VHSV virions have been reported to remain infectious for up to 13 days in water and transport *via* diverse vectors, including boating, ballast water, fishing tackle, and animals, for e.g., amphipod crustaceans, leeches, turtles, and birds (Hawley and Garver, 2008; Bain et al., 2010).

VHSV causes infection in over 140 marine and freshwater species across the Northern Hemisphere and is responsible for finfish diseases worldwide. The World Organization for Animal Health (OIE) reported VHSV as a worldwide fish pathogen (Escobar et al., 2018). Schäperclaus (1938) initially reported viral hemorrhagic septicemia (VHS) disease in Denmark, following mortality observed at Danish rainbow trout farms with infectious kidney swelling and liver degeneration. VHSV has been a major cause of catastrophic losses in European rainbow trout (*Oncorhynchus mykiss*) farms for five decades since the disease was originally described in Germany in the 1930s. In the 1980s, VHSV emerged in the Pacific Northwest region of North America, causing fatal disease in fish in marine and brackish environments of Washington, Alaska, and California. Additional epizootiological investigations in Europe revealed the widespread presence of VHSV in the marine environments of the Baltic Sea, Scotland, the English Channel, Kattegat, Skagerrak, and the North Sea, as well as in Japan and Korea. VHSV has become established in the North American Great Lakes and caused extensive losses to aquafarming. Additional fish mortality episodes appeared during 2006–2008 at several locations in lakes Michigan, Erie, St. Clair, and connected waters (8). The clinical signs and outcome of the infection vary depending on VHSV genotype, fish species, age, stress level, temperature, and other environmental factors (Faisal et al., 2012). VHSV plasticity in terms of host tropism is a matter of concern. This is due to the risk of host jumps from wild fauna to economically important fish species, including the transmission to rainbow trout farms.

VHSV is an enveloped, bullet-shaped, and non-segmented virus. It possesses a single-stranded negative-sense RNA genome comprising 11,158 nucleotides. The linear genome contains six genes encoding a non-virion protein (NV) and five structural proteins, namely, the nucleoprotein (N), phosphoprotein (P), matrix protein (M), glycoprotein (G), and RNA polymerase (L), which are organized as 3-N–P–M–G–NV–L-5 (Schutze et al., 1999). The genes are separated by conserved gene junctions with di-nucleotide gene spacers. The N (nucleocapsid) gene encodes 38–41-kDa proteins that are arranged tightly around the viral RNA genome forming the N–RNA complex, which serves as the template for both transcription and replication. The viral RNA–dependent RNA polymerase consists of two subunits: a large (157–190 kDa) subunit-L and a non-catalytic cofactor, the phosphoprotein P. In addition, P acts as a chaperone of nascent RNA-free N by forming an N (0)–P complex that prevents N from binding to cellular RNAs. The M gene encodes the 19-kDa matrix protein that acts as a bridge between the viral envelope and nucleocapsid in rhabdoviruses and plays a regulatory role in viral transcription, replication, and budding (Faisal et al., 2012). NV, unique to the genus, is capable of suppressing apoptosis at the early stage of viral infection (Ammayappan and Vakharia, 2011), whereas the other five proteins are common in rhabdoviruses with analogous functions (Kurath, 2014).

The 72–80-kDa viral glycoprotein antigen (G-protein), encoded by the *G*-gene, is one of the key components responsible for VHSV cross-species transmission and infection emergence. The *G*-gene has a complete coding length of 1,524 bases, encoding 508 amino acids. The nucleotide sequence encoding the G-protein has been widely used as the preferred molecular marker to assess the VHSV genetic diversity and evolution (Panzarin et al., 2020). Few studies have reported that VHSV-IVb G-gene variants differ with respect to their virulence phenotype (Stepien et al., 2015). The G-protein plays a central role in the host cellular receptor adhesion and insertion. The G-protein is only found in enveloped viruses and makes trimeric spikes that are connected to a lipid bilayer obtained from the host cell membrane. The G-protein is expressed on the surface and plays an antigenic role in triggering significant host immune responses (Jørgensen et al., 1995; Lorenzen et al., 1999). The G-protein is involved in viral emergence and replication. The mutations within the *G*-gene enhance the viral attachment and penetration into the host’s cell and facilitate the virus to evade the host’s immune responses. Phylogenetic analysis based on the nucleotide sequences of *G* and *N* genes of VHSV isolates from marine and freshwater fish species inferred the existence of four genotypes (i.e., I–IV) and several sub-lineages of genotypes I and IV (i.e., Ia–Ie and IVa–IVb) (Einer-Jensen et al., 2004; Nishizawa et al., 2006). The genotype distribution appeared to be geographical-specific rather than host-specific. The genotypes I, II, and III samples are majorly reported to be isolated from Europe, whereas the genotype IV isolates have been sampled from North America and Asia (Kuzmin et al., 2009).

The current study aims to elucidate the genetic distinction and spatiotemporal distribution of VHSV based on *G*-gene. We speculate that VHSV *G*-gene might be under the influence of natural selection, due to its role in the host immune response. Therefore, the possible role of genetic natural selection and recombination, to shape the VHSV genetic structure, was examined. Additionally, the enormous amount of complete *G*-gene sequences available in the public domain repositories from different continental regions motivated us to undertake the current study. The outcomes of this study may implicate in future *G*-gene-based vaccine development against VHSV.

## 2 Materials and methods

### 2.1 *G*-gene sequence retrieval and alignment

The complete *G*-gene sequences of VHSV isolates were retrieved from the virus pathogen resource database of NCBI (Pickett et al., 2012) with known collection date, countries, and host detail information (Supplementary Table S1). The sequence data with no collection date, countries/geography, and host information were excluded according to the strategy of Kim et al. (2016). In addition, the partial CDS sequences of VHSV *G*-gene were excluded as well. Eventually, 710 complete *G*-gene sequences were utilized in downstream analyses. The multiple sequence alignment (MSA) was generated *via* MUSCLE, implemented in MEGA X software (Kumar et al., 2018). The parsimony informative (PI) sites were extracted from the MSA. A total of 577 PI sites were used as an input for population structure, principal component analysis (PCA), and linkage disequilibrium analyses.

### 2.2 Null hypothesis

The linkage disequilibrium state of VHSV was evaluated by testing the null hypothesis of linkage equilibrium using LIAN v3.5 (Haubold and Hudson, 2000). This program employs the Monte Carlo approach (1,000 iterations). The degree of haplotype-wide linkage was estimated by a standardized index of association “I^S^A.” The value of I^S^A is zero in the case of a linkage equilibrium state. Furthermore, two more additional calculations of the linkage disequilibrium (LD), namely, r^2^ and |D′| were computed over the entire alignment using the DnaSP v6.0 program (Rozas et al., 2017). “r^2^” is the variance of the allele frequency between observed and expected haplotypes, whereas |D′| is the measure of the absolute frequency between observed and expected haplotypes (Devlin and Risch, 1995).

### 2.3 Genetic composition analyses

The *G*-gene-based genetic composition of VHSV was investigated *via* STRUCTURE v2.3.4, phylogenetic, and principal component analysis (PCA) approaches.

#### 2.3.1 STRUCTURE analysis

The Bayesian model-based clustering approach, implemented in STRUCTURE v2.3.4 (Pritchard et al., 2000; Evanno et al., 2005), was used to infer the genetic composition and admixture in the VHSV samples based on *G*-gene. This clustering program assigns individuals probabilistically to one or more populations based on allele frequency at each locus (Hubisz et al., 2009). The STRUCTURE algorithms were set to run with different combinations of burn-in periods and Markov chain Monte Carlo (MCMC) simulations, i.e., 50,000–50,000, 70,000–70,000, and 100,000–100,000 iterations, to confirm the consistency of posterior probabilities. Five independent runs were carried out for each K ranging from 1 to 11 to detect the most likely population (K) number. The most optimal K number was estimated by calculating the delta (ΔK) and likelihood values of Ln Pr(X|K) determined by Evanno et al. (2005) employed in STRUCTURE HARVESTER (Earl and VonHoldt, 2012). The genetic structure of VHSV was analyzed using the admixture model along with the option of correlated allele frequency, while the rest of the parameters were kept at default. This model accounts for the individual having mixed ancestry characterized by a set of allele frequencies in subpopulation due to admixture or shared ancestry.

#### 2.3.2 Validation of the genetic structure hypothesis

The genetic composition results obtained from STRUCTURE v2.3.4 were validated by the Fst (fixation index) value. Arlequin v3.11 was used to calculate the Fst value (Wright, 1949) *via* the AMOVA (Analysis of MOlecular VAriance) method with default parameters and 1,000 permutations.

#### 2.3.3 Phylogenetic analysis

The MSA of 710 *G*-gene sequences of VHSV was used for the generation of the phylogenetic tree based on the neighbor-joining (NJ) method (Saitou and Nei, 1987). In addition, the maximum likelihood (ML) tree was reconstructed by the IQ-TREE resource using the general time-reversible + I + G nucleotide substitution model (Trifinopoulos et al., 2016). The infectious hematopoietic necrosis virus (IHNV) glycoprotein gene sequence (Accession ID L40883.1) was used as an out-group. The bootstrap analysis was performed by sampling 1,000 replicates to estimate the accuracy of the phylogenetic tree. FigTree v1.4.4 software was used to visualize the trees (Rambaut, 2018). The tree collapsed at nodes with bootstrap values less than 70%.

#### 2.3.4 Principal component analysis (PCA)

PCA is a multivariate statistical method employed to explore the relationships between variables and samples. PCA is a powerful approach to reduce the dimensionality of complex datasets (Xu and Yuille, 1995; Tharwat, 2016). The approach is commonly used to study the clustering information of samples based on genetic composition. The greatest variance represented by any projection of the data comes to lie on the first coordinate, called the first principal component (PC), the second greatest variance is on the second PC, and so on. PCA was implemented for the dataset of 710 isolates using the PLINK 1.9 resource (Chang et al., 2015), and the plot was visualized *via* the prcomp (stats) function in R.

### 2.4 Recombination analysis

To evaluate the potential recombinants in the VHSV *G*-gene, seven different methods were used in the RDP4 package. These methods are RDP (Martin et al., 2015), Chimera (Posada and Crandall, 2001), GENECONV (Padidam et al., 1999), MaxChi (Smith, 1992), SiSCAN (Gibbs et al., 2000), 3SEQ (Boni et al., 2007), and BOOTSCAN (Martin et al., 2005). A recombination event detected by at least three methods with a *p*-value < 0.01 was considered significant.

### 2.5 Natural selection footprint analysis

The natural selection footprints across the VHSV *G*-gene data were examined by several methods. MSA was subjected to the GUIDANCE server to remove the unreliable alignment region to ensure the alignment accuracy prior to selection analysis (Miller et al., 2011; Privman et al., 2012). The Datamonkey server was used with a default *p*-value = 0.05 to identify the positive and negative selection sites (Pond and Frost, 2005a; Pond and Muse, 2005b). The automatic model selection tool at the Datamonkey server was used to find the best nucleotide substitution model for codon datasets. The GTR (general time-reversible) model was identified as the best nucleotide substitution model for the VHSV *G*-gene datasets. The mixed effects model of evolution (MEME) method (Murrell et al., 2012) was employed to find episodic positive diversifying selection (sites that only affect a few lineages, whereas most are under negative selection) and to estimate the dN/dS value (non-synonymous to synonymous substitution) (Kosakovsky Pond et al., 2006).

### 2.6 Bayesian skyline analysis

To depict changes in the effective population size in VHSV with respect to time based on *G*-gene, a Bayesian skyline plot (BSP) model (Drummond et al., 2005), implemented in the BEAST2 package (Bouckaert et al., 2014), was used. The jModelTest program was performed using the CIPRES server to prioritize the best site substitution model (Miller et al., 2011). The GTR was eventually prioritized as the suitable nucleotide substitution model. Path sampling (PS) and stepping stone (SS) analyses were followed to determine the best clock model *via* Beast1 v1.10.4 by computing marginal likelihood values. The relax clock model was selected as the best model to examine the population size of VHSV. The MCMC steps with a chain length of 300 million generations and log of every 1,000 steps were run. The convergence of all the parameters was determined by the Tracer v1.7.1 program (Rambaut et al., 2013).

## 3 Results

### 3.1 Linkage disequilibrium analysis

To study the population structure of VHSV *via* the STRUCTURE program, the data need to be tested for the hypothesis of whether the loci are in a linkage equilibrium state. The standardized index of association I^S^A value obtained for VHSV was 0.0000 (*p* < 10^−4^, 10,000 replicates), indicating a signal of weak linkage disequilibrium (non-random associations at different polymorphic sites). In order to further investigate the existence of the LD, *|D′|* and r^2^ were calculated. D*′* is the function of LD measurement. The average value of *|D′|* and r^2^ were 0.8783 and 0.0627, respectively, indicating the existence of low LD and weakly linked polymeric loci. This justifies the usage of STRUCTURE to examine the genetic structure of VHSV data.

### 3.2 Population structure and admixture identification

**FIGURE 1:**
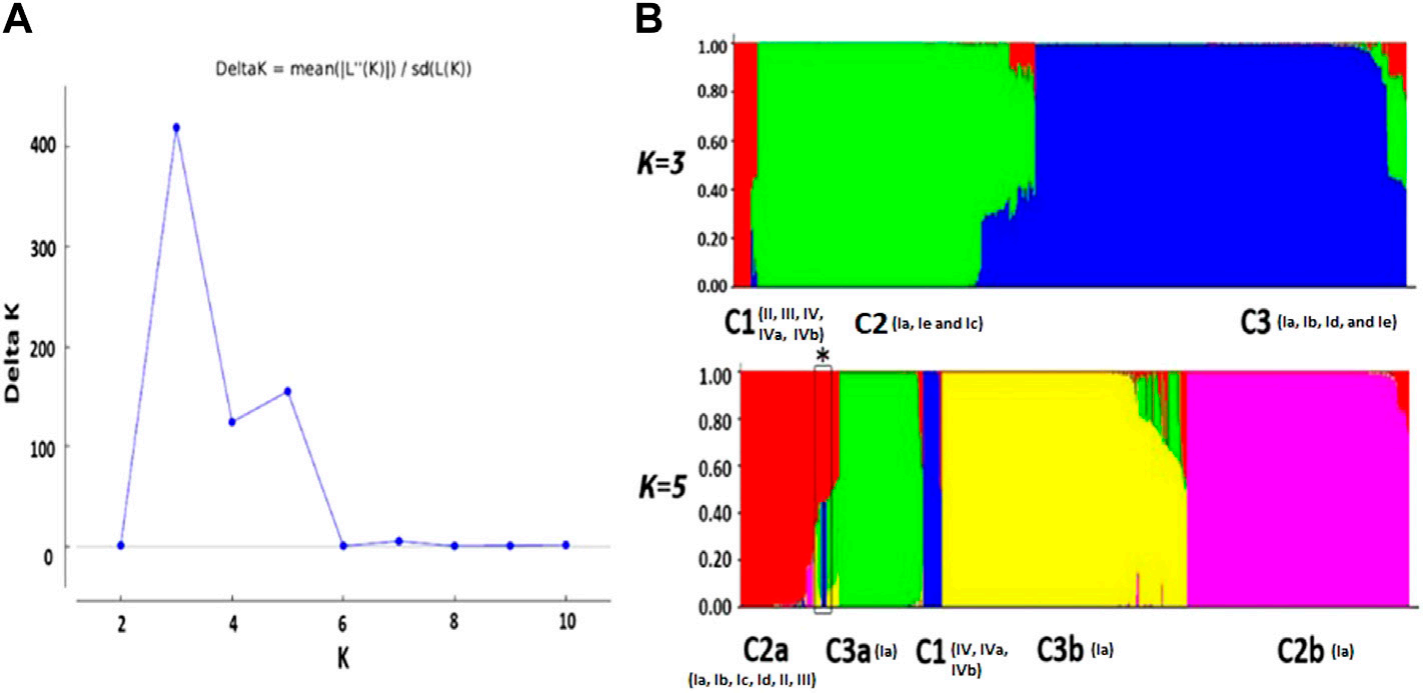
Bayesian structure analysis. **(A)** Plot K vs. ΔK shows the optimum number of subpopulations. K represents the number of clusters. The plot represents the first major peak at K = 3, followed by the minor peak at K = 5 **(B)**. The color bar plots show the genetic structure of VHSV at K = 3 and K = 5 using the admixture model of the STRUCTURE resource. The number in brackets represents genotypes. At K = 3, C1 comprised samples from genotypes II, III, IV, IVa, and IVb. C2 includes genotypes Ia, Ie, and Ic isolated from Denmark, while the genotype Ia samples isolated from other European countries along with genotypes Ib, Id, and Ie are grouped into C3. At K = 5, there are C2a (comprised genotypes Ia, Ib, Ic, Id, II, and III), C3a (genotype Ia), C1 (genotypes IV, IVa, and IVb), C3b (genotype Ia), and C2b (genotype Ia) clusters. Admixed strains were also found in C2a and C3a showing multiple membership scores (marked with a box and *).

The population structure and admixture of VHSV were analyzed *via* the admixture model implemented in the STRUCTURE v2.3.4 program. Five independent simulation runs were pursued for each distinct *K* (i.e., 1–11) to identify the *K*_*opt*_ value (highest peak in the plot *K* vs*. ΔK*). The analysis inferred a major peak at K = 3 and a minor peak at K = 5 (Figure 1A). This clues in toward an index of the hierarchical genetic structure for data of VHSV samples. The minor peak at K = 5 fluctuates across K = 7 and K = 10 during analyses conducted at 50,000–50,000, 70,000–70,000, and 100,000–100,000 burn-ins and burn lengths MCMC simulations. However, a significant major peak at K = 3 stabilized from 50,000 up to the highest, i.e., 100,000 MCMC simulation. The presence of the first major peak K = 3 indicates the primary subdivision of VHSV into three clusters (C1, C2, and C3) (Figure 1B).

Cluster C1 comprises samples from genotypes II, III, IV, IVa, and IVb. Among the members of C1, the genotypes IV, IVa, and IVb majorly are of Asian origin, while the samples from genotypes II and III have been isolated from Europe. Cluster C2 corresponds to members of genotypes Ia and Ic. All the samples from genotypes Ia and Ic isolated from Denmark are grouped in cluster C2, except a Turkish isolate that belongs to genotype Ie, i.e., observed as admixture, holding membership scores of 0.422 and 0.378 from C2 and C3 clusters, respectively. The third cluster, i.e., C3, holds samples from Ia, Ib, Id, and Ie genotypes. The negligible genetic distinction in C3 among different genotype samples might be due to low sample sizes of Ib, Id, and Ie genotypes. The genetic component distinction at *K*_*opt*_ = 3 was additionally confirmed by the high Fst value, i.e., 0.60249 (*p*-value = 0.0000), acquired for the three major clusters. This suggests the statistically promising subpopulation genetic structure existence among the VHSV isolates.

The peak at K = 5 unveils additional diversification of VHSV into five genetic components (C1, C2a, C2b, C3a, and C3b) (Figure 1B). The members of C2 at K = 5 were further stratified into two lineages, namely, C2a and C2b. C2a comprised samples from genotypes Ia, Ib, Ic, Id, II, and III, mostly isolated from rainbow trout. The stratification of genotype 1a samples was observed to undergo time-dependent subdivisions because the genotype 1a samples acquired from 1962 to 1996 were separately clustered in C2a, while cluster C2b corresponds to the genotype Ia samples majorly isolated after 1996 till 2009. Correspondingly, the members of C3 split into two subpopulations, namely, C3a and C3b, both containing genotype Ia samples. The members of the C3 cluster are mostly isolated from trout. At K = 5, C1 comprises genotype IV samples, including IVa and IVb, while the samples from genotypes II and III clustered with C2a members. C1 majorly comprised VHSV samples isolated from the fish host. The Bayesian-based population genetic structure analysis of VHSV *G*-gene observed several admixed strains from subpopulation C2a at K = 5, holding major membership scores from C1a and minor membership scores from C3a ancestries. Additionally, these admixed strains were observed to constitute a distinct clade in the phylogenetic tree.

### 3.3 Phylogenetic analysis of VHSV

**FIGURE 2:**
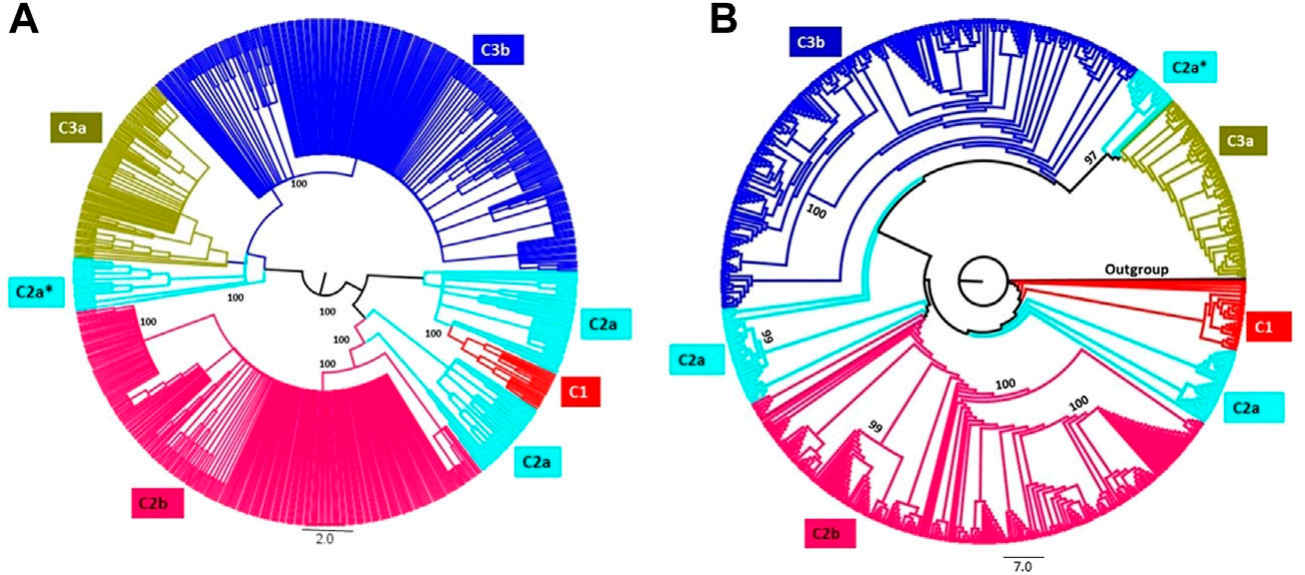
Phylogenetic tree analyses of VHSV isolates. The phylogenetic tree was constructed based on the analysis of VHSV *G*-gene using the neighbor-joining (NJ) **(A)** and maximum likelihood (ML) **(B)** methods. The clades of the tree are colored according to the STRUCTURE result at K = 5. The admixed strains are represented with C2a*. The bootstrap values for 1,000 replicates are shown in bold characters for major branches. The branches of the tree are colored to represent the five subpopulation clusters.

Phylogenetic analysis was carried out for *G*-sequences (*n* = 710) to construct NJ and ML trees with 1,000 replicates. The tree topologies were compared with the clustering pattern acquired from STRUCTURE (at K = 3 and 5). The preliminary delineated K = 3 and K = 5 results assented with the phylogenetic tree (Figures 2A, B; Supplementary Figure S1), except for the C2a subpopulation, which split into three clusters in the phylogenetic tree. The admixed strains identified in the C2a subpopulation during STRUCTURE analysis were characterized to form an independent clade, i.e., C2a* with a significant bootstrap value in the phylogenetic tree (Figures 2A, B). The genotypes Ie, Ib, Ic, II, and III from cluster C2a were clustered closely with cluster C1 (genotypes IVa and IVb). The phylogenetic tree analysis somehow supported the spatiotemporal subdivision of VHSV into subpopulations with additional minor clades within the C3b and C2b subpopulations.

### 3.4 Principal component analysis

**FIGURE 3:**
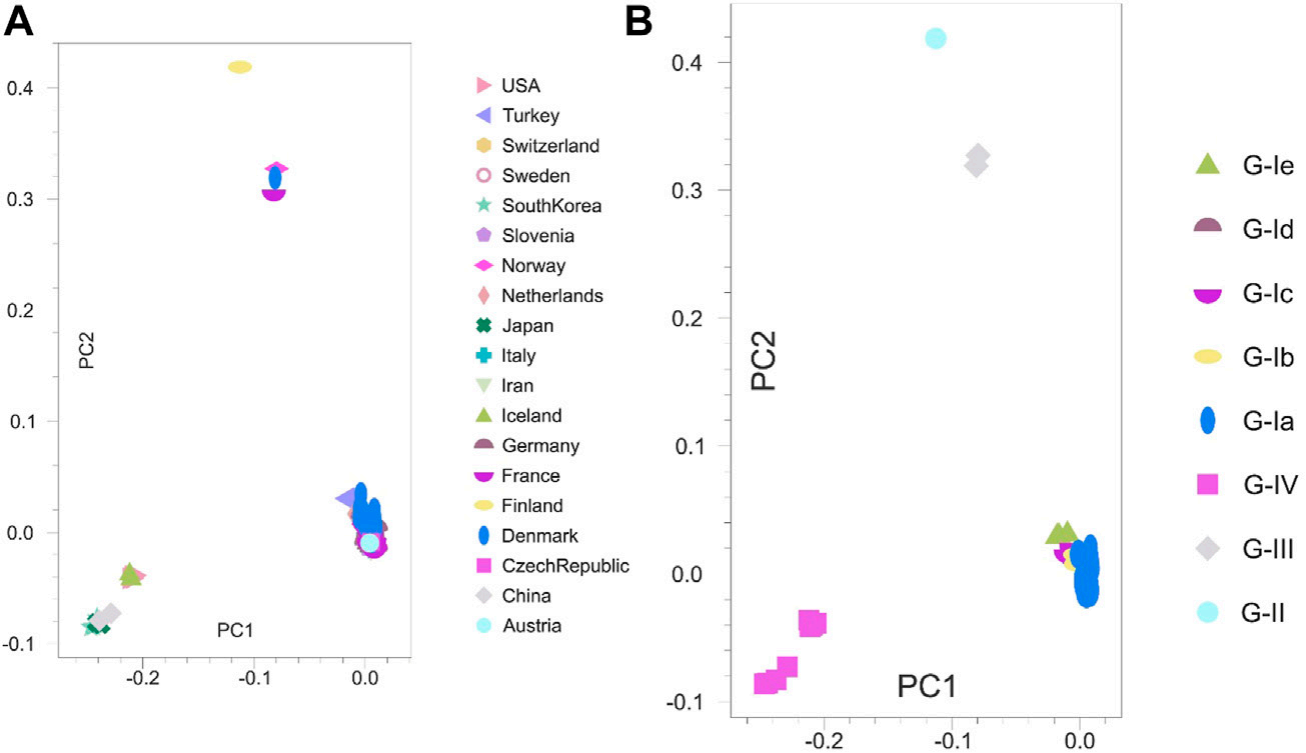
Principal component analysis (PCA). Two-dimensional PCA of VHSV *G*-gene. **(A)** Clustering of VHSV based on geography and country origin from where the samples are isolated. The Asian and United States samples together were observed to constitute a separate cluster at the left side from the European samples. **(B)** Genetic distinction of VHSV samples based on the reported genotypes. The genotype G-IV samples depict marked genetic distinction from the rest of the genotypes.

PCA was pursued to examine the spatial distribution of VHSV samples. The analysis split the Asian and European samples into two PCs (PC1 = 15.72% and PC2 = 14.71%). However, the United States samples clustered closely with the Asian samples (Figure 3A). The genotype-based genetic distinction of VHSV samples was also examined during PCA. The samples from genotypes Ia and Ib tended to appear on the right side of the plot at PC1 (14.98%), whereas the samples from genotypes IV and IVb appeared on the left side of the plot at PC2 (10.71%) (Figure 3B). The PCA findings were majorly congruent with the STRUCTURE and phylogenetic tree patterns.

### 3.5 Pairwise genetic analysis

**FIGURE 4:**
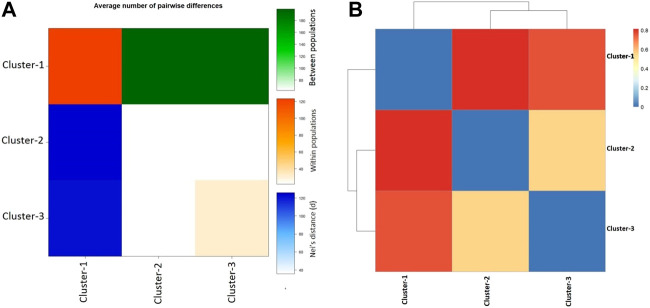
Pairwise population genetic analyses. **(A)** Graph represents the average number of pairwise differences (πxy) between subpopulation clusters (green, above diagonal), within-population clusters *π* (orange, diagonal), and the net number of nucleotide differences between proposed population’s clusters (Nei distance DA) (blue, below diagonal) based on VHSV *G*-gene variants. **(B)** Heatmap plot of pairwise F_ST_ among the three subpopulation clusters of VHSV samples based on *G*-gene.

Additional population genetic structure analysis was performed in the framework of molecular variance analysis (i.e., AMOVA). AMOVA calculates the genetic variance at single or multiple loci due to variation within the population as well as between populations. The locus-by-locus AMOVA analysis inferred that 60.25% of genetic diversity in VHSV samples was attributed due to differences among the population groups (*p* < 0.00001) (Table 1). A higher variance component was observed among population groups than within population groups (Table 1). The significance of AMOVA was estimated by 99,224 permutations. The analyses eventually inferred that genetic diversity in samples mainly arose because of distinct genetic makeup and differentiation among genotypes I, II, III, and IV. The high genetic differentiation was identified within cluster C1, which was comprised of the samples from genotypes IVa and IVb. However, less genetic difference within population groups was observed for the cluster 2 and cluster 3 samples, while the C3 samples showed moderate diversity (Figure 4A). The highest pairwise net number of nucleotide differences (*DA*) and mean pairwise differences (π_xy_) between C1 (genotypes IVa and IVb) and C2 (genotype Ia) were observed among the proposed population’s clusters, whereas the least pairwise net number of nucleotide differences (*DA*) and mean pairwise differences (π_xy_) were observed between C2 (genotype Ia) and C3 (genotypes Ia, Ib**,** Ie, and Id). The highest within-population genetic differentiation (*π*) was observed for C1 samples belonging to genotypes IVa and IVb, while the least *π* value was calculated for the samples from genotypes Ia and Ib (i.e., C2 and C3) (Figure 4A). During pairwise Fst analysis, higher genetic differentiation (Fst = 0.81343) was observed between C1, which was comprised of samples from genotypes II, III, IV, IVa, and IVb, and C2 (genotype Ia). Likewise, high genetic diversity (Fst = 0.76411) was also observed between C1 (genotypes IVa and IVb) and C3 (genotypes Ia, Ib, Ie, and Id), whereas comparatively less genetic differentiation (Fst = 0.56077) was observed between C2 (genotype Ia) and C3 (genotype Ia, Ib, Ie, and Id) clusters (Figure 4B).

**TABLE 1:** Analysis of molecular variance based on *G*-gene sequences of VHSV.

Source of variation	Degree of freedom	Sum of squares deviation	Variance component	Percentage of variation	*p*-value
Among populations	2	8813.897	23.60614 Va	60.25	<0.00001
Within populations	707	11011.188	15.57452 Vb	39.75	<0.00001

### 3.6 Recombination analysis

There was no recombinant VHSV isolate identified based on *G*-gene sequences at a significant *p*-value = 0.00001 threshold. The analyses were repeated with loose *p*-value thresholds, i.e., 0.0001, 0.001, 0.01, 0.1, and 0.5; however, no recombinant sequences were identified. Later on, 50 complete genome sequences of VHSV were examined for recombination events based on set criteria with a *p*-value = 0.00001 threshold. Among these, only two potential recombinant VHSV isolates were identified. The same two recombinant isolates were acquired during subsequent analyses performed at loose *p*-value thresholds, i.e., 0.0001, 0.001, 0.01, 0.1, and 0.5 for complete VHSV genome data. A potential recombinant sample (VHSV-I*), with accession number MK598848, was isolated from China according to the database sample collection record. This recombinant isolate is speculated to have a major parent (accession number MN038328.1) and minor parent (accession number MN038333.1) reported to be isolated from Japan and France, respectively. Another potential recombinant isolate (VHSV-II*), submitted under accession number KC778774, is isolated from Denmark, having a major parent with accession number MN856666 from France and a minor parent with accession number KY359356 isolated from the United States.

### 3.7 Differential selection pressure across the distinct VHSV clusters

The three datasets were prepared for selection scan analysis, according to the three observed genetic clusters. The MEME analysis unveiled five codons with a significant signature of episodic positive selection (*p* = <0.05) for C1 samples, three codons for C2 samples, and eight codons for C3 samples (Table 2). Codon 477 codes for leucine, where synonymous amino acid substitution occurs in all sample sequences grouped in cluster 2. Likewise, codons 58 and 481 code for Arg and Leu, respectively, and synonymous substitution occurs at these codon positions in C3 samples. Multiple amino acid substitutions were observed at the shared codon 258 site (i.e., Glu, Gly, Thr, Ala, Val, and Arg) for samples from clusters C2 and C3 (Table 3). Both C2 and C3 have codon 258 underlined selection, and the amino acid substitutions are mostly the same at this codon site in both of these clusters. However, in the case of cluster 2, a lysine substitution occurs with valine, whereas in the case of cluster 3 samples, the lysine is substituted with arginine and reflects cluster-specific amino acid substitution at this codon site. In addition, multiple amino acid substitutions were observed at positions 212 (i.e., Asn, Glu, and Thr) and 259 (i.e., Lys, Asn, Ala, Glu, Ser, Thr, and Val) in cluster 2 and cluster 3 samples, respectively, portraying the strong selection signal at these respective codon sites. The remaining positively selected codons were identified specific to the dataset of respective clusters and indicate evolutionary distinction or differential selection features among the three proposed genetic clusters of VHSV samples. This unveiled that VHSV sample clusters, identified in the current study, are genetically as well as evolutionarily distinct, and the differential natural selection might have shaped the VHSV genetic diversity. The amino acid sites (aa212, aa258, and aa259) are located within the central region of the G-protein hosting major neutralizing epitopes (Bearzotti et al., 1995). These codon sites are identified underline selection in samples from genotypes Ia, Ib, Ic, Id, and Ie, clumped together in C2 and C3. The selection signature at these sites may arise due to host immune escape that enables the viral particles to infect different host species.

**TABLE 2:** Positive selection codon sites of VHSV *G*-gene based on the MEME method (*p* < 0.05). The false discovery rate controlled by the “*p*-value” and “q-value.”

Codon	Α	β−	Pr [β = β-]	β+	Pr [β = β+]	*p*-value	q-value
Structural gene (*G*-gene)							
C1 (III, IV, IVa, and IVb)							
36	0	0	0.766486	22.6278	0.233514	0.007233	1
60	0	0	0.716969	15.9474	0.283031	0.046233	1
103	1.1737	0	0.924106	238.184	0.075894	0.011557	1
380	2.39702	0	0.919751	278.791	0.080249	0.02659	1
431	0	0	0.759055	29.7144	0.240945	0.011881	1
C2 (Ia and Ic)							
212	0	0	1.00E-09	4.7855	1	0.030555	1
258	0	0	0.63253	54.3082	0.36747	1.70E-05	0.008634
477	0.967826	0	0.956807	54.9306	0.043193	0.024818	1
C3 (Ia, Ib, Ie, and Id)							
58	0	0	0.983773	311.533	0.016227	0.000819	0.207565
258	0.797857	0.797857	0.658363	22.0949	0.341637	0.002918	0.369798
259	2.90919	0.149817	0.428172	22.562	0.571828	0.009962	1
284	0	0	0.222284	5.47083	0.777716	0.020042	1
371	0	0	0.97802	149.86	0.02198	0.049426	1
373	0	0	1.00E-09	3.08805	1	0.033142	1
379	0	0	0.994074	157.695	0.005926	0.001894	0.320096
481	0	0	0.996714	10000	0.003286	7.04E-05	0.035676

**TABLE 3:** MEME method-based episodic selection sites in VHSV *G*-gene among the three subpopulation clusters.

Gene	Protein coded by the gene	Population cluster	Codons sites underlined positive selection	Coding amino acid	Substitution
*G*-gene	Glycoprotein	Cluster 1 (25 sequences)	36	Ala	Val and Pro
			60	Ala	Asp and Ser
			103	Arg	Lys
			380	Thr	Ile
			431	Pro	Leu
		Cluster 2 (293 sequences)	212	Lys	Asn, Glu, and Thr
			258	Lys	Glu, Gly, Thr, Ala, and Val
			477	Leu	Leu
		Cluster 3 (392 sequences)	58	Arg	Arg
			258	Lys	Glu, Gly, Thr, Ala and Arg
			259	Asp	Lys, Asn, Ala, Glu, Ser, Thr, and Val
			284	Lys	Glu and Arg
			371	Ser	Arg
			373	Gln	Lys and Arg
			379	Asn	Gly
			481	Leu	Leu

### 3.8 Bayesian skyline plot analysis

**FIGURE 5:**
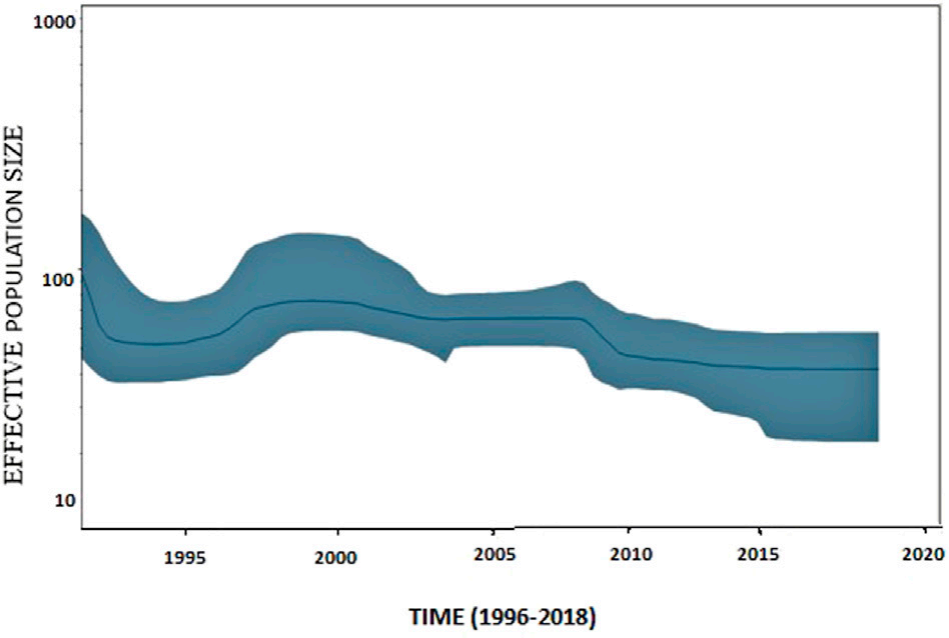
Bayesian skyline plot of VHSV based on *G*-gene features. Bayesian skyline plot (BSP) illustrates the variation in VHSV effective population size over time. The effective population size (Ne) is represented on the y-axis, and generation time is represented on the x-axis in years. A blue line represents the mean effective population size. The 95% HPD (highest posterior density) of genetic diversity estimations is depicted by the shaded blue area.

The *G*-gene sequence data of VHSV, deposited in a public database, spanned over approximately two decades. The Bayesian skyline plot analysis of the entire data depicted a gradual decrease in VHSV population size from 2008 to 2009; however, later on, the plot displayed an undeviating pattern of variation from 2010 to 2018. A slight increase in variation in VHSV population size was observed from 1996 to 2000 (Figure 5).

## 4 Discussion

The comprehensive *G*-gene-based population genetic analyses were performed to infer the genetic diversity and evolutionary distinction across the VHSV samples. The methodological approach employed in the current study has previously been adopted for other viruses, including *Begomovirus* (Prasanna et al., 2010), coxsackievirus (Khan and Khan, 2021), *Lassa Mammarenavirus* (Forni and Sironi, 2020), rhinovirus (Waman et al., 2014), and human norovirus (Kakakhel et al., 2022). The analyses speculate that spatiotemporal and selection pressure events possibly contribute toward the diversification of the VHSV isolate samples deposited in the public database.

The current study was pursued with VHSV samples belonging to four genotypes. The model-based Bayesian analysis initially stratified VHSV isolates into three main subpopulations. The analysis inferred that genotype Ia was stratified into two clusters, namely, C2 and C3, while genotypes II, III, and IV clumped together in C1 (Figure 1A). Genotype Ia isolates from Denmark were clustered independently in C2, while genotype Ia samples acquired from Germany, Italy, Switzerland, Iran, Turkey, and Japan were clustered in C3. Genotype Ia indicates distinction based on geography. The distinctive subpopulation feature of European freshwater VHSV genotype Ia isolates is formerly reported by Kahns et al. (2012), where Ia-1 was reported to specifically infect the Danish freshwater catchments, while Ia-2 originates from other European countries. The genotype Ia VHSV samples from rainbow trout, clustered in C2 in this study, may have evolved within the Danish water catchments that contain a high number of infected rainbow trout farms. The Danish watersheds, infected with VHSV, are considered a geographically isolated area separated from the rest of the country region. However, few VHSV samples from Denmark appeared to cluster within C3 during the current analysis. These VHSV isolates might have been introduced into Denmark by the accidental transmission of infected samples from other European countries. The VHSV transmission among these states might have occurred due to failure of biosecurity measures, for e.g., improper disinfection of many fish transport lorries. Additionally, angling is a popular tourist activity in Denmark, where there are numerous “put and take lakes”, stocked with domesticated fish species. The transmission of disease might have occurred due to the use of a contaminated fishing gear or an infected bait. Moreover, there was a common practice of feeding farmed fish with marine species. It was suspected that marine fish species are responsible for the widespread transmission of VHSV into the freshwater ecosystem. Therefore, when marine fish feeding to rainbow trout was prohibited in Denmark in 1985, the number of infected farms declined, and this led to the successful control of VHS in Denmark (Skall et al., 2005).

The analyses of the current study unveiled that all the Iranian VHSV isolates acquired from freshwater rainbow trout farmed clumped within cluster C3, i.e., majorly constituted by the VHSV samples collected from other European countries. VHSV may get introduced in Iran due to infection transmission from European regions. The 9 *G*-gene sequences of Iranian VHSV samples isolated from trout belonged to genotype Ia-2, the same as the VHSV isolates reported from trout farms of European countries. Previous investigations inferred that the VHSV genotypes of European origin might be introduced in Iran through imported eyed eggs. The annual demand of 300–400 million eyed eggs for the Iranian aquaculture industries is fulfilled *via* more than 70% of imports from European countries. However, due to the economic and political situations, this import is presumably not under proper veterinary control. Iranian fish farms are tightly interconnected and exchange eggs, small fry, and fish, thus providing an opportunity for the spread of viruses across the sectors. This frequent exchange of viruses between farms might be the main reason for the close identity of the Iranian isolates with each other (Ahmadivand et al., 2021).

C1, majorly constituted by the genotype IV isolates, is reported to cause infection in a wide range of host species and invade fish species in both freshwater and marine environments in North America (Escobar et al., 2018). At K = 5 (Figure 1B), the analysis unveiled the diversification of VHSV into five subpopulations, and the genotype Ia samples isolated from Denmark were observed to undergo time-dependent clustering. The highest within-group genetic heterogeneity was identified for C1 samples in the current analyses, and additional lineages may emerge in the future from genotype IV specimens. The PCA and phylogenetic analysis also supported the aforementioned information. Additionally, many minor lineages were observed in the case of genotype Ia samples during phylogenetic analysis which might cause additional outbreaks in future.

Fewer recombination events were evident among VHSV samples in the current study. Recombination is reported to occur less frequently in negative single-stranded RNA viruses (RNA) compared to positive single-stranded RNA viruses (RNA^+^) (Chare et al., 2003). Patiño-Galindo et al. (2021) recently reported a significant inverse association between the genome size and the frequency of recombination events. The study provides a rationale for fewer recombination events in a large VHSV genome, i.e., 11 kb, compared to other viruses, including hepatitis B and C. Additionally, the current recombination results are consistent with the prior findings of the limited recombination events in the Rhabdoviridae family (Davis et al., 2005).

The BSP analysis of VHSV isolates in the current study inferred that the effective population size of VHSV slightly increased approximately from 1996 to 2000 (Figure 5). The increase in effective population during 1996–2000 coincided with the diversification of genotype 1a into two clusters, namely, C2a and C2b, as identified in the STRUCTURE-based analysis. Cieslak et al. (2016) investigated the BSP of VHSV genotype 1a samples using entire *G*-gene sequences and observed a substantial increase in population size during the years 2000–2001 and 2001–2003 (Cieslak et al., 2016), which contradicts the findings of the current study. These discrepancies might be due to variations in the isolate sequence datasets, i.e., Cieslak et al. (2016) only examined genotype Ia of European origin.

Evidence of the diversifying genetic structure in VHSV led us to understand the role of episodic positive selection pressure to shape its genetic composition. The positive selection scan analysis in the current study unveiled sixteen codon sites of the *G*-gene that exhibit the significant signature of positive selection. The study by He et al. (2014) also reported a mean ratio of dN/dS < 1.0 per site for the six VHSV genes. Schonherz et al. (2018) reported underline positive selection in six amino acid sites (6, 212, 258, 259, 505, and 506). Three amino acid positions (aa212, aa258, and aa259) are congruent with the results of the current study. In addition, these three amino acid sites are reported to locate in close proximity to two of the six disulfide bonds detected in the VHSV G-protein (Einer-Jensen et al., 1998). These disulfide bonds are involved in the structural conformation of the neutralizing epitope. Amino acid changes close to either of the two disulfide bonds might affect the bonding properties and potentially cause the conformational changes of the neutralizing epitope. With respect to observed clusters in the current analysis, codon site 212 is identified underline selection specifically in cluster 2, whereas codon site 259 selection feature is specific to cluster C3 samples, thus deciphering evolutionary distinction among clusters. However, a positive selection signature at codon site 258 is observed in both C2 and C3 samples because this site plays a significant role in the epitopic region in the coding protein. The substitution at positively selected amino acid sites, namely, aa212, aa258, and aa259 might enable the VHSV to either directly escape neutralizing antibodies or to provide the mutants with a selective advantage by facilitating the escape from the host immune responses. The current analysis identified three positive selected codons, namely, 58, 481, and 477 in the G-gene sequences of all the VHSV isolates undertaken in the current study. These codons might be promising during vaccine target prioritization.

Evidence of differential selection across codon sites of VHSV *G*-gene was identified in the three respective subpopulation clusters of VHSV. This differential selection in samples may arise due to differential environmental circumstances or host specificity. This implies that VHSV’s emergence in different hosts is accompanied by adaptive evolution in the G-protein to facilitate cross-species transmission. The five positive selection codons, namely, 36, 60, 103, 380, and 431 were found specific to the dataset of cluster 1, which was majorly comprised of samples from genotypes IVa and IVb. Genetic differentiation in the genotype IV samples allowed the virus to persist over time, albeit at lower levels and with less virulence (Stepien and Niner, 2020). The ongoing evolutionary differentiation of genotype IV may likely facilitate its potential to spread and adapt, in future, to new habitats and non-acclimated hosts. The three positive selected G-gene codons, namely, 212, 258, 477 were majorly observed in the cluster C2 dataset, with one codon, namely, 258 shared by C2 and C3. Likewise, the codon 212 and 477 sites are unique to the C2 samples. The seven episodic positive selection codons, namely, 58, 259, 284, 371, 373, 379, and 481 were specific to the C3 dataset.

Additionally, the signature of negative selection was observed in the VHSV *G*-gene with a higher frequency of the synonymous substitutions than the non-synonymous (dN/dS < 1) substitution. This suggests purifying selection as the principal evolutionary force acting upon the VHSV *G*-gene. In general, positive selection sites may responsible for the immune pressure, leading to an escape mutation, and maintain the sites that increase virus fitness, whereas negative selection or purifying selection is responsible for removing mutations that are lethal and cause reduced fitness. Epitopes (short peptides) are the minimal structural feature recognized by the immune system and are the principal components of subunit vaccines. Characterizing the positive selection features of the pathogen proteins holding epitopic features is valuable to identify vaccine candidates worthy of experimental validation. The sites evolving under strong purifying selection may suggest targets for vaccine or antiviral development with improved resistance profiles. However, identifying sites evolving under negative selection is challenging, as it is often impossible to distinguish from sampling bias. Therefore, designing the vaccines based on the information on positive selected sites is more appropriate than that on negative selection sites (Domingo et al., 2007; Dolan et al., 2018; Goodswen et al., 2018). DNA vaccination, based on expressing the viral membrane glycoproteins, has proven to be one of the effective strategies for inducing a protective immune response in rainbow trout under experimental conditions against the VHS and other rhabdoviruses (Anderson et al., 1996; Lorenzen et al., 2000; LaPatra et al., 2001). However, no vaccines are commercially available so far against VHSV. The antigen-encoding DNA vaccine for fish immunization has been approved against IHNV and commercially used in Canada (Evensen and Leong, 2013). The *G*-gene-based DNA vaccination strategy could be promising against VHSV. However, the genetic distinction and homogeneity of this gene in different continental regions, as addressed in the current study, might be considered to devise an effective vaccination strategy worldwide.

## 5 Conclusion

The population hierarchical study of the VHSV was pursued on their available complete *G*-gene sequences. The isolates were initially clustered into three subpopulations based on genetic heterogeneity, congruent with the clustering pattern of phylogenetic tree analysis. The VHSV samples were additionally stratified into five subpopulations at K = 5, speculating the possible emergence of new VHSV genotypes and lineages. The genetic structure analyses inferred that the majority of genotype Ia isolates collected from Denmark constituted a separate cluster. Later on, these samples were clustered into two lineages. The evidence of episodic positive selection events was observed in VHSV *G*-gene. Some of the positive selection sites were located within the conformation-dependent neutralizing epitope regions of the coding protein, suggesting an adaptation to immune response-related genetic variation across different host fish species. The analyses of the current study unveiled that most of the observed genetic diversification is based on mutation rather than recombination. The current analyses unveiled that genetic mutation might be an important factor driving observed selection as well as genetic stratification among the VHSV samples. The molecular epidemiological information may implicate in the prevention and control of VHSV infection in aquafarms.

## Data Availability

The datasets presented in this study can be found in online repositories. The names of the repository/repositories and accession number(s) can be found in the article/Supplementary Material.
