# Supplementary figures and images for: Population genetic analyses unveiled genetic stratification and differential natural selection signatures across the G-gene of viral hemorrhagic septicemia virus
Source: Front Genet. 2022 Dec 12;13:982527. doi: 10.3389/fgene.2022.982527 (PMC9790968; doi:10.3389/fgene.2022.982527)

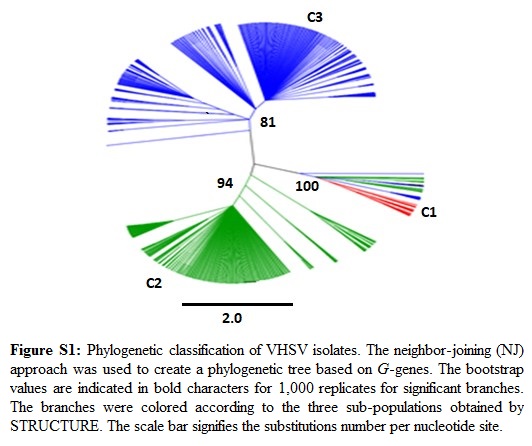

Supplement: Supplementary file 1 [file Image1.JPEG]
